# Hitting the Target! Challenges and Opportunities for TGF-β Inhibition for the Treatment of Cardiac fibrosis

**DOI:** 10.3390/ph17030267

**Published:** 2024-02-20

**Authors:** Maria Vistnes

**Affiliations:** 1 Department of Cardiology, Oslo University Hospital Ullevål, 0450 Oslo, Norway; mariavistnes@gmail.com; 2 Institute of Clinical Medicine, University of Oslo, 0450 Oslo, Norway

**Keywords:** heart failure, cardiac fibrosis, TGF-β, anti-fibrotic therapy

## Abstract

Developing effective anti-fibrotic therapies for heart diseases holds the potential to address unmet needs in several cardiac conditions, including heart failure with preserved ejection fraction, hypertrophic cardiomyopathy, and cardiotoxicity induced by cancer therapy. The inhibition of the primary fibrotic regulator, transforming growth factor (TGF) β, represents an efficient strategy for mitigating fibrosis in preclinical models. However, translating these findings into clinical benefits faces challenges due to potential adverse effects stemming from TGF-β’s physiological actions in inflammation and tissue homeostasis. Various strategies exist for inhibiting TGF-β, each associated with a distinct risk of adverse effects. Targeting TGF-β directly or through its signaling pathway proves efficient in reducing fibrosis. However, direct TGF-β blockade may lead to uncontrolled inflammation, especially following myocardial infarction, while interference with the signaling pathway may compromise structural integrity, resulting in issues like insufficient wound healing or ventricular dilatation. Influencing TGF-β activity through interacting signaling pathways, for instance by inhibitors of the renin–angiotensin–aldosterone-system, is insufficiently potent in reducing fibrosis. Targeting activators of latent TGF-β, including ADAMTS enzymes, thrombospondin, and integrins, emerges as a potentially safer strategy to reduce TGF-β-induced fibrosis but it requires the identification of appropriate targets. Encouragement is drawn from promising agents developed for fibrosis in other organs, fueling hope for similar breakthroughs in treating cardiac fibrosis. Such advances depend on overcoming obstacles for the implementation of anti-fibrotic strategies in patients with heart disease, including fibrosis quantification. In this review, insights garnered from interventional and mechanistic studies, obtained through a non-systemic search spanning preclinical and clinical evidence, are summarized to pinpoint the most promising targets for further exploration and development.

## 1. The Need for Anti-Fibrotic Therapies in Heart Failure Management

**Figure 1:**
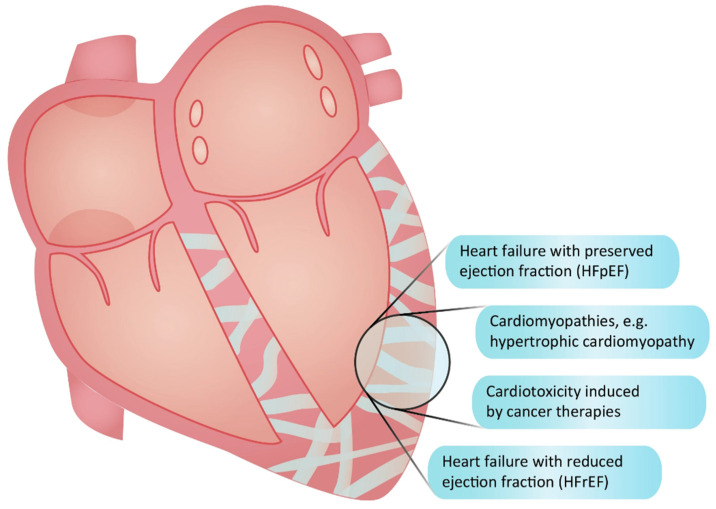
Conditions characterized by cardiac fibrosis.

Fibrosis denotes an excessive accumulation of extracellular matrix (ECM) components, such as collagens, deposited by activated fibroblasts [1]. Present in most cardiac diseases (Figure 1), fibrosis is linked to the deterioration of cardiac function, heightened susceptibility to arrhythmia, increased symptomatology, and inferior outcomes [2,3]. Various stimuli, including inflammation, neurohormonal activation, mechanical stress, aging, and toxic insults, act synergistically to drive cardiac fibrosis, resulting in increased cardiac stiffness and diastolic dysfunction [4,5,6]. A subsequent rise in filling pressures and congestion are central characteristics of heart failure with preserved ejection fraction (HFpEF), a condition where fibrosis is a pivotal yet inadequately treated pathological hallmark [7]. Despite the recent introduction of sodium-glucose-cotransporter-2 (SGLT2) inhibitors [8], there remains a substantial unmet need for novel treatments, specifically anti-fibrotic drugs, capable of enhancing outcomes for HFpEF patients [9]. This need, however, may be less pronounced in patients with heart failure with reduced ejection fraction (HFrEF). Although cardiac fibrosis is also present in HFrEF patients [10], their heart may exhibit less fibrosis than that observed in HFpEF [11]. In HFrEF, fibrosis primarily results from cardiomyocyte loss contributing to systolic dysfunction. While anti-fibrotic therapies are potentially beneficial in this context, other drugs with effect on systolic function are already available [12]. Additionally, beyond the quantitative and functional aspects of fibrosis, differences in the location, composition, and organization of the fibrotic ECM exist between the two types of heart failure [13]. Consequently, the development of anti-fibrotic therapies should be tailored specifically to the type of heart failure being addressed, with a potentially higher likelihood of additive effects to existing therapies in patients with HFpEF.

## 2. The Significance of Fibrosis as a Therapeutic Target in Cardiomyopathies and Cardiotoxicities

Cardiac fibrosis constitutes a pathogenic factor in various heart diseases, exemplified by hypertrophic cardiomyopathy and cardiotoxicity induced by cancer treatments (Figure 1). In hypertrophic cardiomyopathy, fibrosis serves as an early disease manifestation driven by the activity of transforming growth factor (TGF)-β [14]. Fibrosis is detectable in a majority of the patients [15], correlating with worsened diastolic function [16], ventricular arrhytmias [17], the clinical progression of the disease [18], and an increased risk of sudden cardiac death [19,20,21,22], with a 30% rise in mortality risk for every 10% increase in fibrosis volume [23]. Although novel myosin inhibitors show promise in ameliorating symptoms and cardiac dysfunction for hypertrophic cardiomyopathy patients [24], they fall short in reducing fibrosis after the phenotype has been established [25]. Given that hypertrophic cardiomyopathy stands as the leading cause of sudden death in young individuals [26], the search for additional treatment options becomes imperative. Cardiotoxicity encompasses adverse myocardial changes, including fibrosis, triggered by chemotherapy and radiation [27]. Anthracyclines and radiation are known instigators for fibrosis development, inducing myocardial injury and inflammation or exerting direct effects on TGF-β [28]. Anti-fibrotic therapies present a potential avenue to counteract these detrimental effects on the myocardium during cancer therapy. Beyond these examples, cardiac fibrosis contributes to a diverse array of cardiac diseases, where anti-fibrotic therapies could be employed to delay disease progression and improve clinical outcomes.

Figure 1 shows examples of conditions in the heart where fibrosis is involved (figure created by Vistnes).

## 3. The Dual Nature of Fibrosis and TGF-β in the Heart: Adaptive and Maladaptive Roles

Fibrosis, often presenting as a deleterious process with negative implications, also exhibits an adaptive and indispensable facet. An illustrative example of this adaptive role is reparative fibrosis where the formation of a scar to replace dead cardiomyocytes after infarction becomes crucial for maintaining tissue integrity. Conversely, maladaptive fibrosis manifests as an exaggerated wound healing response to persistent or recurrent pathological stimuli [1,7]. This process is fueled by persistently activated fibroblasts that have evaded apoptosis [7,29]. Maladaptive fibrosis can be diffusely spread in the ECM in response to systemic pathological factors, termed reactive fibrosis, or localized around vessels, as observed in hypertension, referred to as perivascular fibrosis [6,7,11]. The intricate nature of these diverse fibrotic processes in heart disease presents challenges in generating effective anti-fibrotic strategies. At the core of cardiac fibrosis regulation is TGF-β, which is implicated in all types of fibrosis [30,31,32,33], with variations in isoform expression corresponding to the stage of fibrosis [34]. Beyond its role in fibrosis, TGF-β plays a pivotal role in homeostatic functions, including inflammation resolution [31] and tissue integrity maintenance [35]. The significance of TGF-β in tissue homeostasis is underscored by the uncontrolled inflammation observed in mice with a genetic deletion of Tgfb1 [36], and the aneurysmal formation in patients with mutations in the TGF-β receptor genes, as seen in Loeys–Dietz syndrome [37]. These instances highlight the need for a nuanced approach to targeting fibrotic regulators based on our understanding of the purpose of fibrosis and the pathological and physiological processes governed by TGF-β. The pursuit of novel and safe therapies necessitates judicious target selection, precise intervention timing, and tailored approaches for specific patient populations. 

## 4. Targeting Activators of Latent TGF-β for Selective Inhibition

**Figure 2:**
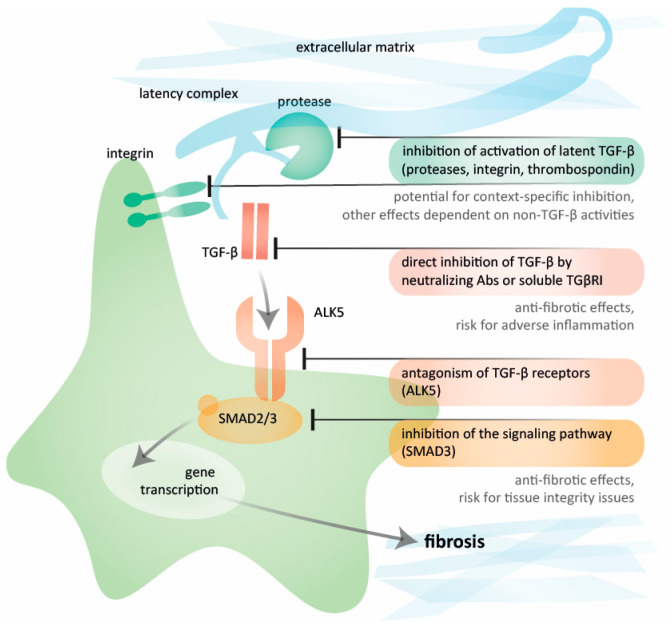
Potential targets for TGF-β inhibition. Different levels of inhibiting TGF-β-induced fibrosis with some examples of therapeutic strategies, including the inhibition of activation of latent TGF-β by integrins or proteases, the direct blockade of TGF-β by neutralizing antibodies and soluble TβRII, inhibitors of ALK (also termed TβRI), and the inhibition of signaling pathways. Figure created by Vistnes.

Given the multi-level regulation of TGF-β activity, several targets within the pathway for TGF-β activation and signaling are amenable to pharmaceutical modulation (Figure 2). These targets encompass factors involved in the activation of TGF-β denoting the release of the mature TGF-β dimer that facilitates binding to its receptor. The stores of latent TGF-β provide an opportunity for cell-specific activation based on stimuli and available activators [38,39,40]. Initially secreted as a small latency complex alongside latency-associated peptide (LAP), this small latency complex is bound to the latent TGF-β binding protein 1 (LTBP1) forming the large latency complex [41,42]. This latency complex is anchored to ECM proteins like fibrillin microfibrils [43] or fibronectin [44], exhibiting strong binding to an isoform called EDA-fibronectin found in fibrotic tissues [45]. The activation of latent TGF-β can occur through integrin-mediated conformational changes [46], protease-induced release [39,47,48], binding to the matricellular protein thrombospondin-1 [49], or to GARP (glycoprotein-A repetitions predominant protein) [50]. Mechanical stress and inflammatory mediators act as stressors triggering activation through the induction of activating factors [38,39,51]. The identification of tissue- and context-specific activators offers an opportunity for the targeted inhibition of TGF-β activity. However, the relevant targets may possess properties beyond impact on TGF-β activation, contributing to an overall treatment effect. 

## 5. Inhibiting TGF-β Directly: Targets in the Signaling Pathway

Upon activation, TGF-β modulation can occur through the direct inhibition of the mature dimer, receptor antagonism, or the inhibition of downstream signaling pathways. These strategies target TGF-β-signaling across various tissues and conditions. Briefly, the activated TGF-β dimer binds to the TGF-β receptor (TβR) II and then TβRI, also known as ALK5. This binding triggers the phosphorylation of transcription factors SMAD2 and SMAD3, which, in conjugation with SMAD4, translocate into the fibroblast nucleus to induce the transcription of fibrotic components such as collagens. Furthermore, the activation of the pathway promotes the transition into activated myofibroblasts [52]. The target genes depend on the presence of co-regulators that can associate and collaborate with SMAD complexes to regulate transcription, potentially adapting gene transcription based on tissue type and environmental cues [53,54]. In addition, the three isoforms of TGF-β carry distinct temporal expression patterns, which may allow for tailoring of inhibitory actions with isoform-specific agents. Furthermore, non-canonical pathways involving Erk1/2, JNK, Rho GTPases, p38 MAP kinase, Src tyrosine kinase, and phosphatidylinositol (PI3) kinase may also be activated [55,56], offering alternative strategies to modulate TGF-β signaling. Understanding the biological roles of the components within the signaling pathway lays the foundation for developing TGF-β signaling inhibitors while mitigating potential toxicity associated with extensive TGF-β inhibition.

## 6. Direct Blockade of TGF-β: Timing Matters!

The direct blockade of TGF-β, which inhibits the interaction of the TGF-β dimer with its receptor, has demonstrated astonishing efficacy in reducing fibrosis in animal models. However, caution is warranted as this approach may have detrimental effects by interfering with the physiological roles of TGF-β [57]. Indeed, pan-TGF-β inhibition utilizing agents that target all three isoforms of TGF-β is associated with inflammation and the degeneration of coronary arteries and heart valves [58]. The dual nature of TGF-β inhibition has been highlighted through gene therapy to elevate soluble TβRII or the use of neutralizing TGF-β antibodies in mice, also emphasizing the critical role of timing in the context of myocardial infarction. Administering such treatment before coronary ligation increased mortality, worsened left ventricular dilatation and systolic function, and exacerbated immune response within the infarcted myocardium [59,60]. Conversely, initiating treatment after the infarction did not impact inflammation, but prevented systolic dysfunction and left ventricular dilatation [59]. No observable effects were noted when the treatment commenced later, at 4 weeks after infarction [61]. Throughout these time points, TGF-β blockade consistently reduced fibrosis in the non-infarcted myocardium without affecting infarct size [59,61]. In situations where adaptive fibrotic responses are less critical, such as in rats subjected to pressure overload, neutralizing antibodies of TGF-β have shown beneficial effects by preventing fibrosis and improving diastolic dysfunction [62]. Similarly, LAP peptides inhibiting the interaction between TGF-β and its receptor have been shown to decrease fibrosis, attenuate the activation of the TGF-β signaling pathway, and prevent left ventricular dilatation and dysfunction in mice subjected to isoproterenol exposure [63]. However, the translational potential for direct TGF-β blockade is unclear, as the direct TGF-β blockade with the monoclonal antibody fresolimumab has been associated with adverse effects like keratoacanthomas in clinical trials for non-cardiac indications [64]. To summarize, while the direct inhibition of TGF-β holds potential in reducing cardiac fibrosis, the possibility of adverse effects like inflammation and the intricate timing considerations limit its applicability. 

## 7. Balancing Efficacy and Adverse Effects of Inhibitors of TGF-β Receptors

Several inhibitors have been developed for TGF-β receptors, particularly for ALK5, effectively disrupting the canonical signaling pathway downstream of TGF-β. The anti-fibrotic potential of ALK5 inhibitors, such as GW788388 and galunisertib, has been substantiated in preclinical models of non-cardiac fibrosis [65,66]. Encouragingly, similar positive outcomes are observed in animal models of heart disease. In a mouse model of myocardial infarction, the application of the ALK5 inhibitor GW788388 from a week after infarction attenuated the development of fibrosis, systolic dysfunction, and left ventricular dilatation [67]. Notably, the same agent exhibited promising results in a mice model of Chagas cardiomyopathy, where it reduced fibrosis, improved cardiac function, and lowered the risk of arrhythmia [68]. Some of these benefits may stem from indirect systemic effects, since a reduction in the activation of the renin–angiotensin–aldosterone system has been observed by another ALK5 inhibitor, SD-208 [69]. ALK5 may escape detrimental effects on inflammation as observed for direct TGF-β blockers, although this is largely undetermined as the ALK5 inhibitors are not tested during the critical phase of infarct healing. However, ALK5 inhibitors are associated with heart valve lesions [70] and impaired tissue regeneration. Indeed, in a study using the ALK5 inhibitor SM16 in rats subjected to aortic banding, the treatment efficiently reduced fibrosis, but resulted in left ventricular dilatation and increased mortality due to the rupture of the aortic banding site [71]. Similarly, mutations in TβRII prevented fibrosis but promoted ventricular dilatation and dysfunction in response to aortic banding in mice [72]. These observations underscore the necessity for more selective targets to circumvent unwanted effects on TGF-β-mediated tissue regeneration and integrity. 

## 8. SMAD Deficiency and Loss of Structural Integrity

While direct inhibitors of TGF-β and ALK5 broadly impede TGF-β-induced mechanisms, targeting the SMADs within the canonical pathway may offer a more focused approach given the more limited homeostatic properties of SMAD2 and SMAD3 [73,74]. In mice, the genetic deletion of Smad3 protected against the post-infarction development of systolic dysfunction, fibrosis, left ventricular dilatation, and ruptures during the first week [75,76]. These observations imply that selectively targeting SMADs could reduce maladaptive fibrosis without compromising their advantageous roles in the early immune responses following infarction [77]. Furthermore, the fibroblast-specific deletion of Smad3 greatly reduced fibrosis in response to aortic banding [30], and Smad3 deficiency protected against fibrosis in diabetic mice [78]. However, targeting SMAD3 may introduce other potential adverse effects. Notably, Smad3 deletion in mice has been associated with an elevated risk of late ruptures after infarction [79] and with aortic dilatation and spontaneous aortic rupture in diabetes [78]. Moreover, the cardiomyocyte-specific loss of Smad4 led to a dilated cardiomyopathy phenotype characterized by detrimental remodeling and heart failure [80]. Collectively, these loss-of-function studies suggest that the inhibition of SMADs may carry risks related to the loss of structural integrity in both cardiac and non-cardiac tissues. However, a more moderate reduction in SMAD3 through pharmacological inhibition has not been thoroughly explored and could potentially yield more tolerable effects than those observed in knockout studies. Moreover, in a study where a negative transcription factor downstream of TGF-β was overexpressed, reduced fibrosis and the preservation of left ventricular dimensions and function were observed following myocardial infarction in rats [81]. This observation suggests that other strategies to interfere with the signaling pathway may yield more tolerable results.

## 9. The Challenges of Targeting MMPs in Heart Disease

Matrix metalloproteases (MMPs) are potential therapeutic targets in heart diseases due to their effects on ECM degradation, cell migration, and inflammation [82,83,84,85]. Due to their activation of latent TGF-β, MMP-2 [86], MMP-9 [87], and MT1-MMP (MMP-14) [88,89] harbor potential as targets for mitigating TGF-β-inducible fibrosis. However, in mice treated with selective inhibitors of MMP-2 or MMP-9 initiated on the first day after myocardial infarction, a delayed resolution of inflammation was observed, without discernible effects on subsequent cardiac remodeling or fibrosis development [90,91]. The potential anti-fibrotic effects of MMP inhibitors may be more apparent in other conditions. For instance, in mice subjected to doxorubicin-induced cardiotoxicity, MMP inhibition demonstrated anti-fibrotic effects by restricting MMP-2 activity [92]. The genetic deletion of MT1-MMP in mice protected against fibrosis upon pressure overload [93], but this potential therapeutic target has not been explored by pharmaceutical intervention. Despite promising results in preclinical studies, the translation of MMP inhibitors to clinical testing has encountered challenges. The selective inhibition of MMPs triggers compensatory increases in other MMPs [85], potentially explaining the disparate effects on inflammation observed when inhibiting versus deleting MMP-9 [82,94]. On the other hand, the insufficient selectivity of MMP inhibition has been linked to adverse effects, such as musculoskeletal syndrome, imposing limitations on dosing in clinical trials [95,96]. A phase II trial with a broad-acting MMP inhibitor, initially promising in animal models [95], selected suboptimal doses and failed to demonstrate effects on post-infarction remodeling in patients with STEMI [96]. However, recent advancement in chemical structures may have addressed concerns related to insufficient selectivity [97]. Coupled with an improved understanding of the biological effects of different MMPs, new attempts to test MMP inhibition for reducing cardiac fibrosis in appropriate preclinical models can be justified. 

## 10. Enhancing Precision in Integrin Antagonism for the Prevention of Latent TGF-β Activation

Integrins are transmembrane receptors connecting the ECM to the intracellular cytoskeleton [98], where a subset are capable of activating latent TGF-β, rendering them potential targets for anti-fibrotic therapies. Integrins containing the αV subunit can activate latent TGF-β by binding to an Arg-Gly-Asp (RGD) motif on LAP, inducing conformational changes that release the mature TGF-β [98,99,100], in some situations in collaboration with proteases [101]. Notably, αVβ1 and αVβ6, found on fibroblasts, play a specific role in TGF-β-induced fibrosis development [99]. Despite their significance, there are currently no approved drugs specifically targeting αV-integrins [98]. Challenges in previous drug discovery programs stemmed from agonist effects exerted by antagonist molecules, as conformational changes could induce a shift from a low-affinity to a high-affinity state. In recent years, improved strategies have successfully addressed these challenges, paving the way for the development of novel integrin inhibitors for fibrotic conditions [102]. Noteworthy candidates such as PLN-74809, GSK-3008348, and STX-100 have progressed to phase I and phase II trials [103]. However, achieving narrow selectivity remains challenging, and most integrin inhibitors will target a group of integrins [102]. While the most advanced drug candidates have not been tested in cardiac fibrosis, earlier integrin inhibitors, such as the integrin subunit αv inhibitor cilenglitide, have demonstrated proof-of-principle for the beneficial effects of integrin inhibition in heart disease. Cilenglitide prevented immune cell infiltration and the transition to heart failure in mice with hypertrophic cardiomyopathy [104]. Moreover, cilenglitide reduced infarct size, diminished fibrosis, improved systolic function, and decreased mortality in mice with myocardial infarction [105]. These findings suggest that promising integrin inhibitors, with demonstrated anti-fibrotic effects in other organs, may hold promise in alleviating cardiac fibrosis.

## 11. Targeted Inhibition through Other Activators of Latent TGF-β

In the pursuit of strategies to counter the activation of TGF-β, inhibiting ADAMTS4 (A Disintegrin And Metalloprotease with Thrombospondin Motif 4) or thrombospondin-1 emerges as a promising approach. Unlike the constitutive expression of several MMPs, ADAMTS4 exhibits low expression in normal tissues but substantially increases in response to pathological stimuli and various disease states [39,51,106]. ADAMTS4 is associated with fibrosis in the heart [39], lung [107], and kidney [108], implicated in early inflammatory reactions to pathological stimuli [107]. By cleaving EDA-fibronectin, ADAMTS4 enhances the bioavailability of TGF-β [39]. The pharmacological inhibition of ADAMTS4, either through a selective inhibitor or the broader-acting inhibitor pentosan polysulfate, has shown promise in preventing heart failure development following aortic banding in rats [51]. Selective inhibition resulted in reduced fibrosis, improved survival rates, and preserved diastolic and systolic function [39]. Therefore, targeting the ADAMTS4-mediated release of latent TGF-β presents a potentially safe approach to inhibiting TGF-β, avoiding interference with its actions in normal physiology. This notion is supported by the observed high tolerability of pentosan polysulfate [109]. Thrombospondin-1 is a known activator of TGF-β [49], but also plays diverse roles in hemostasis, angiogenesis, cell adhesion, migration, nitric oxide signaling, and the regulation of other growth factors. Binding between a KRFK sequence in thrombospondin-1 and an LSKL sequence in LAP disrupts the interactions between TGF-β and LAP, leading to the exposure of the sequences that bind to the receptor [110]. Blocking the interaction between thrombospondin-1 and LAP with an LSKL peptide reversed cardiac fibrosis, improved left ventricular function, and reduced TGF-β activity in hypertensive diabetic rats [111]. Further exploration of the roles of activators like ADAMTS4 and thrombospondin-1 in TGF-β fibrosis could validate their potential as therapeutic targets. 

## 12. TGF-β-Suppressing Agents with Unknown Mechanisms of Action

Anti-fibrotic agents where the precise target is unknown represent additional potential strategies for reducing TGF-β activity. Pirfenidone, an approved treatment for idiopathic lung fibrosis, reduces TGF-β signaling *in vivo* and *in vitro* I [112]. Administering pirfenidone in mice from week 1 to 5 after myocardial infarction preserved systolic function, diminished fibrosis, and restrained infarct size [113]. In the PIROUETTE phase II trial, pirfenidone reduced fibrosis in patients with HFpEF and pre-existing fibrosis as demonstrated using magnetic resonance imaging, but an effect on diastolic dysfunction was not detected [114]. This treatment is associated with manageable adverse effects like nausea, dizziness, and insomnia [115]. Although the treatment effect was modest, the PIROUETTE trial provides guidance and hope to future studies employing anti-fibrotic therapies in HFpEF patients. Tranilast, a tryptophan metabolite with an unknown mechanism of action, represents another TGF-β-suppressor [116] that reduces fibrosis in rats with diabetes and hypertension [117]. In addition, numerous natural products have demonstrated inhibitory effects on fibrosis and TGF-β signaling in experimental models [118,119,120,121]. Moreover, the inhibition of specific molecular players, previously unrecognized in TGF-β activation processes, has shown efficacy in reducing both fibrosis and TGF-β activity [122,123], suggesting that new targets for TGF-β inhibition can be identified as further mechanistic studies unfold. Beneficial effects are also observed for inhibitors of non-canonical pathways, exemplified by the inhibitor of p38 MAPK signaling by RWJ-67657 which showed improved systolic function, less fibrosis and left ventricular dilatation, as well as smaller infarct size when initiating the treatment after the challenging period of active infarct healing [124]. Additionally, the membrane ionophore salinomycin, identified through a high-throughput screen for inhibitors of myofibroblast activation, has been shown to decrease fibrosis and suppress p38 MAPK signaling in animal models [125]. While these agents demonstrate potential in inhibiting fibrosis in heart disease, therapies with more direct effects on TGF-β may have more robust effects on cardiac fibrosis.

## 13. Through the Backdoor: TGF-β Modulation via the Renin–Angiotensin–Aldosterone System

Current heart failure therapies exhibit anti-fibrotic effects either by indirectly targeting fibrosis-promoting factors or through interactions among signaling pathways [126]. Renin–angiotensin–aldosterone system (RAAS) inhibitors, such as ACE inhibitors, angiotensin II receptor antagonists, and aldosterone antagonists, are part of the recommended treatment regimen for HFrEF [12]. Angiotensin II and aldosterone stimulate fibrosis by intertwining with the TGF-β pathway through crosstalk between their respective signaling pathways [127,128]. Preclinical models and clinical studies have demonstrated the anti-fibrotic efficacy of RAAS inhibitors. For instance, in mice overexpressing TGF-β1, the angiotensin II receptor antagonist telmisartan reduced cardiac fibrosis [129]. In patients with hypertrophic cardiomyopathy [130], and hypertension [131], one-year treatment with the angiotensin II receptor antagonist losartan attenuated fibrosis progression. Similarly, a six-month course of the ACE inhibitor lisinopril showed reduced fibrosis and improved diastolic function in hypertensive patients compared to antihypertensive treatment with hydrochlorthiazide [132]. The potential for RAAS inhibition to prevent cardiotoxicity induced by cancer treatment such as doxorubicin has been discussed [133], and observations in animal models suggest that such effects may be mediated through the reduction of TGF-β activity and fibrosis [134]. Aldosterone antagonists like finerenone and eplerenone exhibited anti-fibrotic capabilities in murine models [135,136]. In patients, one year of spironolactone treatment ameliorated stiffness and cardiac fibrosis in HFrEF patients with fibrosis [137]. However, the anti-fibrotic effect in patients with HFpEF after six months was less certain [138]. Despite the biological foundation of utilizing RAAS inhibitors to treat TGF-β-mediated fibrosis, the observed anti-fibrotic effects are modest. The limited effect may reflect that the TGF-β pathway remains responsive to other stimulating factors. This notion is supported by the lack of success in phase III clinical trials testing ACE inhibitors [139], angiotensin II receptor antagonists [140], and aldosterone antagonists [141] in HFpEF patients. Consequently, achieving more robust effects on fibrosis may require the addition of therapies with more direct influences on TGF-β.

## 14. Modest Anti-Fibrotic Effects of Other Heart Failure Drugs

Beyond RAAS inhibitors, anti-fibrotic effects are reported for several other drugs employed in heart failure treatment. Patients with HFrEF receive SGLT2 inhibitors, neprilysin inhibitors, and betablockers [13], while SGLT2 inhibitors stand as the sole disease-modifying treatment recommended for HFpEF [8]. In animal models, SGLT2 inhibitors demonstrate effects on TGF-β-mediated fibrosis, including in murine models with cardiac fibrosis induced by angiotensin II infusion [142] or diabetes [143]. The precise mechanisms are poorly understood but may be mediated by indirect effects through dampening the RAAS system and inflammation [144,145]. Studies in preclinical models have also indicated anti-fibrotic effects of neprilysin inhibition. Rats undergoing myocardial infarction [146] or aortic banding showed reduced fibrosis when being treated with a neprilysin inhibitor, possibly through natriuretic peptide receptors interacting with the TGF-β/SMAD pathway [147], although the reproducibility of this effect is not consistent [148]. Betablockers, integral in HFrEF management, lack apparent anti-fibrotic actions [126]. Mice with TGF-β1 overexpression did not show altered fibrosis when treated with the betablocker metoprolol [129], and betablockers are not proven to be beneficial in HFpEF patients [149]. Among loop diuretics used for symptom relief, torasemide demonstrated the reduction of fibrosis and improvement in left ventricular function in hypertensive heart disease, albeit through an unknown mechanism [150]. Despite these observations, currently available heart failure medications do not exert sufficient effects on mitigating the detrimental impact of fibrosis, justifying the search for novel agents to reduce cardiac fibrosis. 

## 15. Challenges in Translating Anti-Fibrotic Strategies from Other Organs to the Heart

While few anti-fibrotic agents have been advanced to clinical testing for heart disease, there is a richer clinical pipeline for other fibrotic indications [1]. There are several factors that may explain why the development of agents for cardiac fibrosis has fallen behind. The continuous wear and tear during the cardiac cycle in the heart makes anti-fibrotic treatment more challenging as the loss of ECM integrity can result in cardiac rupture after myocardial infarction or left ventricular dilatation during pressure overload. Adverse effects, which might be tolerable in conditions with a higher symptom burden, such as idiopathic lung fibrosis, could be unacceptable for patient populations with fewer symptoms as in early phases of HFpEF and in hypertrophic cardiomyopathy. Moreover, the heterogeneity among patients with cardiac fibrosis, particularly those falling under the category of HFpEF, highlights a need to categorize the patients into better defined populations [151] and to detect fibrosis in a feasible and non-invasively manner. Measuring the extracellular volume using late gadolinium enhancement in magnetic resonance imaging has emerged as a reliable method for fibrosis quantification in clinical trials [114], and could be exploited to select patients and monitor treatment effects in future trials on anti-fibrotic drugs. If these obstacles could be addressed, important advancements may also be made in the field of cardiac fibrosis.

## 16. Conclusions and Future Directions

There is a need for developing anti-fibrotic drugs for use in heart disease, and TGF-β represents an important target, being the critical regulator of most fibrotic processes. Previous studies have demonstrated that anti-fibrotic effects can be achieved, but that interference with physiological properties of TGF-β, particularly the resolution of inflammation and the maintenance of structural integrity, poses challenges in developing strategies that are both safe and efficient. There is hope that drugs currently under development for fibrosis in other organs may also be efficient in cardiac fibrosis. Activators of latent TGF-β identified in mechanistic studies may represent a group of targets with higher potential for achieving the safe and effective inhibition of TGF-β-mediated fibrosis, as these mechanisms can be specific to tissue and context. 
